# Book review of *Shaping the African Savanna* by Michael Bollig

**DOI:** 10.1186/s13570-021-00215-3

**Published:** 2022-03-01

**Authors:** M. Timm Hoffman

**Affiliations:** Department of Biological Sciences, University of Cape Town, Private Bag X7, Rondebosch, 7700 South Africa

**Keywords:** Colonialism, Communal rangelands, Conservation, Environmental history, Governance, Wildlife

## Abstract

Bollig, M, *Shaping the African Savannah: from Capitalist frontier to Arid Eden in Namibia.* Cambridge: Cambridge University Press; 2020. 404 pages, hardback, ISBN 9781108488488

The African Studies Series of Cambridge University Press has produced several influential works about the continent, and this book by Michael Bollig is among the best. *Shaping the African Savannah: From Capitalist Frontier to Arid Eden in Namibia* is a contribution of extraordinary depth and breadth and is one of the most comprehensive regional environmental histories I have read. Bollig (2020) describes the 150-year history of the Kaokoland region in north-western Namibia in exquisite detail drawing on a wide range of archival sources, oral histories, published and unpublished literature and field data collected over 25 years of research in the region. One of the main contributions of the book is to show how Kaokoland has undergone distinct phases that have changed at multi-decadal time scales. Numerous influences incorporating social and political violence, regional and international capital inflows and technological innovation, such as the installation of boreholes, legal and policy changes, mega-droughts, have all ruptured the wider social-ecological system with knock-on effects for the ecological infrastructure and pastoralists of the region. Bollig reminds us that it helps to know where you are in the spatio-temporal scale of one’s own work to be able to fully comprehend these impacts and to assess their influence and meaning.

The book is over 400 pages and is an absorbing read primarily because of its clear, systematic organisation and simple, well-written text, which is largely devoid of jargon. It also helps that Bollig presents a summary at the start of each of the main sections which outlines the main themes covered in that section. The only thing I missed in the book was a detailed map of the region. Place names of towns, rivers, mountains and springs were often mentioned in the text, and knowing something of the geography of Kaokoland would have been helpful.

The book is arranged in six parts organised broadly along a temporal sequence from the pre-colonial period to the present. The first part, however, begins with a personal account of how the author became interested in the area and describes his long association with Kaokoland, its environment, social history and people of the region. What started as an analysis of risk management in pastoralist societies in the 1990s grew into a review of the entanglement and interconnectedness of the natural and human history of Kaokoland. Part 2 explores the pre-colonial environments of north-western Namibia, the emergence of pastoralism and the impact that people and elephants had on the ecological infrastructure of Kaokoland in the late nineteenth and early twentieth centuries. Hunting and wide-scale cattle raiding expeditions by colonial settlers and commandos from different tribal associations as well as the rinderpest pandemic of the 1890s resulted in a self-inflicted depastoralisation of the region at the time. Also, as a result of hunting, populations of elephants, rhinos and hippos—the entire megafauna—were completely changed with significant consequences for the environment.

Part 3 focuses on the repastoralisation of the region in the first half of the twentieth century and highlights the impact that colonialism had on pastoralism through the politics of encapsulation and the control of mobility. Laws enacted by the South African government, who administered the region, controlled all aspects of pastoralism including where people settled, where they could move and how they managed the landscape. For example, local people were not allowed to hunt wildlife and were even prevented from setting fires to rejuvenate rangelands. They were also not allowed to utilise the Kunene River, even during droughts. Internal migrations within Kaokoland were discouraged, and trade with their Angolan neighbours was strictly prohibited, ostensibly to control the spread of diseases such as contagious bovine pleuropneumonia. Despite these severe restrictions, and the territorialisation of the pastoral system, cattle numbers increased significantly with a concomitant shift in vegetation cover from perennial to annual grasses under conditions of increased grazing pressure.

Part 4 documents the changes that occurred during the period between the 1950s and 1980s and outlines the impact that the extensive provision of boreholes had on both pastoralism and vegetation structure. It also addresses the role that the war, waged by the South African Defence Force on Namibians, had on the environment, on wildlife and on the people of the region. Wildlife, for example, was nearly hunted out in Kaokoland in the 1960s and 1970s while the war severely disrupted the social relations within families and wider society across the region. The resistance offered by traditional authorities and by pastoralists to the control that the state had on resources as well as their objections to other agricultural interventions, such as the animal breeding schemes, is described in detail. Local people wanted more land and the freedom to exercise their own authority over the region’s resources. Despite these objections, the provision of boreholes, which Bollig describes as nothing short of a hydrological revolution, significantly changed the patterns of pastoral mobility and led to an intensification of herding in which cattle numbers rose fourfold in just two decades. The ensuing transformation of the landscape, and the almost complete breakdown of the pastoral system in the 1980 drought, however, worried conservationists who, through a series of detailed reports, outlined the role that conservation could play in the wider ecology and economy of the region.

This becomes the focus for part 5 where the emergence of the ‘New Commons’, the conservancies, established by the Namibian government with significant international support, is outlined. This section provides one of the most comprehensive evaluations of the conservancy movement in northern Namibia that I have read. It draws on a wide range of published and unpublished sources as well as personal research undertaken in the region to evaluate the role that this initiative has had on the social-ecological environment of Kaokoland. Bollig shows how young people, including younger women, rather than older men, who represent the traditional authorities, have benefitted from the establishment of conservancies across Kaokoland. He completed the book towards the end of 2019, and it would be interesting to read a more recent analysis from him of the impact that the COVID-19 pandemic has had on the conservancies and the communities they supported.

In the final section, part 6, Bollig contextualises his analysis and theorises about how pastoral systems change over space and time. He generalises his findings from Kaokoland and shows how key developments are embedded within a global and regional capitalist system and how the distribution of both internal and external power structures has shaped what has happened to the social-ecological environment. He discusses his results in terms of the adaptive cycle of resilience theory constituted by the four distinct phases of exploitation, conservation, release and reorganisation. He points to the increasing complexity of the social-ecological dynamics in Kaokoland over the 150-year period covered by the book and how it is the ecological infrastructure itself which provides some measure of resilience to the system. He also looks to the future and evaluates some of the different scenarios that have been proposed for Kaokoland and for the country within Namibia’s Vision 2030.

This book is special for several reasons but primarily because of the impressive level of detail contained in each of the chapters. Bollig has provided exhaustive summaries of the archival sources and has systematically covered a wide range of relevant themes in each of the main disciplines. He appears as comfortable with a discussion of rangeland ecology and the quality of perennial and annual grasses as with the role of government grants on household incomes and regional economies or with the influence of international capital on the conservancy model. The ease with which the analysis straddles the disciplines is notable as is its coverage of the issues from multiple points of view. For example, Bollig points to the violence of colonialism and of the South African occupation and the impact they both had on the lives of Herero and Himba pastoralists but also acknowledges the influence of different developments, role players and institutions when appropriate. The most important contribution is to show how natural history and human history are intertwined and how together they have impacted on the lives of pastoralists living in north-western Namibia. I wish I had a book like this in my library 30 years ago. Future environmental historians are fortunate to have this template to work from and to use as a standard against which to evaluate the quality of their own contributions.

## Data Availability

Not applicable.
